# Postoperative cognitive dysfunction: spotlight on light, circadian rhythms, and sleep

**DOI:** 10.3389/fnins.2024.1390216

**Published:** 2024-04-17

**Authors:** Ellie Campbell, Mariana G. Figueiro

**Affiliations:** Light and Health Research Center, Department of Population Health Science and Policy, Icahn School of Medicine at Mount Sinai, New York, NY, United States

**Keywords:** cardiac surgery, circadian rhythm, circadian rhythm disruption, cognitive dysfunction, sleep

## Abstract

Postoperative cognitive dysfunction (POCD) is a neurological disorder characterized by the emergence of cognitive impairment after surgery. A growing body of literature suggests that the onset of POCD is closely tied to circadian rhythm disruption (CRD). Circadian rhythms are patterns of behavioral and physiological change that repeat themselves at approximately, but not exactly, every 24 h. They are entrained to the 24 h day by the daily light–dark cycle. Postoperative CRD affects cognitive function likely by disrupting sleep architecture, which in turn provokes a host of pathological processes including neuroinflammation, blood–brain barrier disturbances, and glymphatic pathway dysfunction. Therefore, to address the pathogenesis of POCD it is first necessary to correct the dysregulated circadian rhythms that often occur in surgical patients. This narrative review summarizes the evidence for CRD as a key contributor to POCD and concludes with a brief discussion of how circadian-effective hospital lighting can be employed to re-entrain stable and robust circadian rhythms in surgical patients.

## 1 Introduction

Common differential diagnoses of postoperative neurological disturbances with impaired cognitive performance include postoperative cognitive dysfunction (POCD), delirium, central anticholinergic syndrome, dementia, and akinetic crisis (Rundshagen, 2014). This narrative review focuses on POCD, which is a common complication of cardiac surgery, characterized by a decline in attention, awareness, and cognitive ability. Highly invasive procedures, such as on-pump coronary artery bypass grafting surgery, are typically associated with an increased risk of POCD (Ge et al., 2014). Up to 50% of patients experience short-term symptoms of POCD following cardiac surgery, and up to 30% of patients report cognitive deficits that can persist more than 6 months after surgery (Tan and Amoako, 2013). POCD is associated with poor postoperative recovery, reduced health-related quality of life, and higher mortality (Rundshagen, 2014).

Advanced age is the primary risk factor for POCD. Age is a strong predictor of cerebrovascular disease and cognitive impairment, which are the very disorders implicated in postoperative neurological complications (Deiner and Silverstein, 2009). Immune priming in the aging brain also elevates the risk of systemic neuroinflammation (Wei et al., 2019). The onset of POCD often overlaps with pre-existing health vulnerabilities in elder populations, resulting in loss of independence and increased caregiver burden (Butz et al., 2019).

A growing body of literature implicates circadian rhythm disruption (CRD) in the pathogenesis of POCD. Circadian rhythms are endogenously driven 24-h oscillations that regulate key physiological functions, such as body temperature and the sleep–wake cycle. These processes are generated by an internal clock located in the suprachiasmatic nucleus (SCN) of the hypothalamus (Herzog et al., 2017). The SCN is entrained to the daily light–dark cycle by retinal light exposures that continuously reset the 24-h circadian pattern to synchrony with the solar day at one’s location on Earth (Duffy and Czeisler, 2009). Accordingly, the SCN also regulates the function of peripheral clocks located in various tissues throughout the body, which modulate the expression of circadian genes in a range of physiological functions (Richards and Gumz, 2012).

As a narrative review, this paper examines a subset of the literature on postoperative CRD and sleep disturbances and how these may be related to POCD. It discusses potential countermeasures, including the use of a modified hospital lighting system to promote circadian entrainment and improve postoperative sleep disturbances. Lighting installations in hospital units are typically designed to maximize visibility for healthcare workers, often at the expense of patients’ sleep quality and circadian stability (Albala et al., 2019). By providing a robust light–dark pattern that promotes entrainment of circadian rhythms, lighting shows promise for mitigating symptoms of POCD and accelerating the recovery of patients undergoing cardiac surgery. Light also has a direct effect on humans. Most notably, it elicits a strong alerting effect on people, like a cup of coffee. Light exposure during the day can not only promote entrainment, but also increase daytime alertness.

This paper focuses primarily on the entraining effects of light and on data collected from older adults (age ≥ 60) undergoing cardiac surgery; studies involving younger and/or non-cardiac patients are also incorporated to illustrate general trends. Searches were conducted on PubMed and Google Scholar, using keywords including “postoperative circadian rhythm disruption,” “postoperative sleep disruption,” “postoperative cognitive dysfunction,” “postoperative neuroinflammation,” and “lighting interventions for patients.”

## 2 The circadian system and the sleep–wake cycle

### 2.1 The circadian system

Almost all organisms experience cyclic physiological and behavioral rhythms. These cyclic rhythms that repeat themselves at approximately every 24 h are regulated by the suprachiasmatic nuclei (SCN) in the hypothalamus (Vitaterna et al., 2001). The function of the SCN as a central pacemaker is well-established, as demonstrated when circadian rhythms were abolished in murine models with SCN lesions (Granados-Fuentes et al., 2004). The SCN is entrained or synchronized to the local time by external factors, known as *zeitgebers* (time givers). The daily light–dark pattern from the retina reaching the SCN is the primary *zeitgeber* for the circadian system (Blume et al., 2019).

SCN neurons contain a network of core clock genes and their translated proteins, which oscillate over a 24-h period to generate circadian rhythms. Several positive and negative feedback loops regulate the function of the circadian oscillator. In particular, the transcription factors CLOCK and BMAL1 heterodimerize to activate transcription of the clock-controlled genes *Period* and *Cryptochrome*. *Period* and *Cryptochrome* proteins accumulate in the cytoplasm, form PER/CRY heterodimers, and translocate to the nucleus to inhibit the activity of CLOCK/BMAL1. As a result, *Period* and *Cryptochrome* transcription is suppressed (Cox and Takahashi, 2019). Clock genes are expressed not only in the SCN but also in peripheral organs and tissues. The SCN entrains and synchronizes gene expression in these peripheral clocks, primarily through oscillations in hormone secretion (Balsalobre, 2002).

### 2.2 The two-process sleep model

Humans are a diurnal species who are awake during the day and asleep at night. The two-process sleep model is currently the most widely accepted model of sleep regulation. It postulates that the sleep–wake cycle is regulated by two key processes that act in opposition throughout the 24-h day: the circadian process (known as Process C) and the sleep–wake dependent homeostatic process (known as Process S). Process C is driven by the circadian system and is synchronized to the diurnal light–dark cycle, whereas Process S is governed by a pressure for sleep that builds during wakefulness and dissipates during sleep. The SCN generates a wake-promoting signal that becomes progressively stronger throughout the day, reaching a peak shortly before bedtime. This wakefulness signal is subsequently lost during secretion of nocturnal melatonin (Dijk and Archer, 2009). Consistent with the idea that the SCN facilitates the initiation and maintenance of wakefulness during the day, Edgar et al. showed that in monkeys who had their SCN lesioned, their total sleep time significantly increased, and sleep–wake, sleep stages, brain temperature, and the circadian rhythm of drinking were eliminated (Edgar et al., 1993).

Adenosine, a byproduct of the hydrolysis of adenosine triphosphate, is a key factor in sleep homeostasis. During the daytime, high levels of metabolic activity in the brain cause adenosine to accumulate. Adenosine then activates A_1_ and A_2A_ receptors to inhibit arousal circuits and promote sleep. This mechanism allows the body to compensate for sleep deprivation by increasing both the duration and depth of subsequent periods of sleep (Deboer, 2018). These two opposing processes combine to regulate wakefulness and sleep propensity in organisms (Deboer, 2018). It should be noted that, although time of day and prior time awake are the two primary factors affecting sleep, other behavioral and environmental factors such as eating, mating, caring for offspring, and illnesses can also affect sleep (Duhart et al., 2023). Clock genes, particularly the PER2 gene, have also been associated with the homeostatic process of sleep. A common variant in PER2 was shown to be associated with a 20-min reduction in slow wave sleep, which is a marker of sleep homeostasis (Chang et al., 2016). Those with a polymorphism in the PER3 clock gene (PER3(5/5)) tend to be morning types and exhibit more rapid build-up of sleep pressure during sleep deprivation. These studies show the close link between clock genes and the sleep–wake cycle.

### 2.3 Effects of sleep deprivation on cognition

Sleep–wake disruption leading to sleep deprivation is associated with various cognitive symptoms that often overlap with changes in brain physiology. A key characteristic of the sleep-deprived brain is a notable decline in attention and focus. Sleep loss has been shown to impair performance on tasks requiring sustained attention or the ability to maintain focus on stimuli presented repeatedly over a long period of time. When presented with such tasks, sleep-deprived individuals will typically display irregular and sporadic periods of focus punctuated by lapses in attention known as “microsleeps” (Massar et al., 2019). Brain imaging studies reveal that this attentional impairment can be attributed to reduced activity in the frontoparietal network (FPN), a group of brain regions involved in executive functioning, coupled with intermittent activation of the default mode network (DMN), the brain’s resting-state network (Krause et al., 2017). The state of imbalance between task-driven and unfocused activity in the sleep-deprived brain results in impaired attention during cognitively demanding tasks.

In addition, sleep deprivation impairs working memory, the ability to retain relevant information for immediate use over a short period of time. Working memory and attention are both key components of executive functioning processes and operate through common neural networks (Zhou et al., 2022). Similarly to attention, working memory is inhibited by reduced FPN activity and inappropriate DMN activation after sleep deprivation (Chee and Choo, 2004). Furthermore, sleep deprivation is associated with deficits in long-term memory, likely due to impaired hippocampal function and synaptic plasticity (Prince et al., 2014). As summarized by Lim and Dinges, sleep deprivation severely affects one’s ability to respond quickly, significantly increases the number of response lapses, and significantly enhances time on a test bout in a psychomotor vigilance task (Lim and Dinges, 2008).

Finally, sleep deprivation has been shown to induce changes in mood and behavior by disrupting the functional connectivity of the amygdala, a component of the limbic system that oversees emotional responses. Sleep-deprived individuals exhibit markedly reduced connectivity between the amygdala and ventral anterior cingulate cortex, resulting in heightened sensitivity to negative stimuli (Motomura et al., 2013). Furthermore, insufficient sleep diminishes the ability of the medial prefrontal cortex to regulate amygdala activity and stabilize emotional responses (Motomura et al., 2017). Sleep deprivation therefore promotes high reactivity and emotional dysregulation and is thought to precipitate disorders such as anxiety and depression (Bauducco et al., 2016).

## 3 Postoperative cognitive dysfunction

### 3.1 Circadian and sleep disruption in cardiac surgery patients

Disturbances in sleep architecture are common among hospitalized patients and manifest as a decline in nocturnal sleep duration and/or quality. Ho et al. found that 36% of inpatients with no history of severe sleep disturbance reported insomnia during their stay in the general medical ward of a community hospital, with 68% of reports citing staff awakenings from periodic blood draws and vital sign checks and 23% citing environmental factors such as ambient noise and light intrusions (Ho et al., 2017). The physiological toll of cardiac surgery amplifies these effects. Liao et al. report that up to 50% of cardiac surgery patients experience sleep disturbance attributable to environmental factors and physiological symptoms such as pain and dyspnea. Sleep disturbance was found to persist even after discharge because of psychological symptoms such as anxiety and fluctuations in mood. For most of those patients, sleep quality did not return to preoperative levels for 2 months (Liao et al., 2011).

Anesthesia has been identified as a factor in postoperative sleep disturbance (Luo et al., 2020). Most general anesthetics target synaptic transmission in the central nervous system, depressing excitatory and enhancing inhibitory transmission (Platholi and Hemmings, 2022). Therefore, ion channel receptors for inhibitory neurotransmitters such as γ-amino butyric acid (GABA) are the primary targets of such anesthetic agents (Garcia et al., 2010). Interestingly, the pharmacologic “sleep” generated by anesthesia shares certain pathways with physiological non-rapid eye movement (NREM) sleep. The ventrolateral preoptic nucleus (VLPO) of the hypothalamus is highly active during NREM sleep and contains primarily GABAergic neurons. Anesthetic drugs promote GABA neuronal activity in the VLPO. Thus, common patterns of neural activation exist between physiological and pharmacologic sleep (Mashour and Hudetz, 2017). These interactions may explain the incidence of sleep disturbance after anesthesia. Anesthetic drugs have been shown to increase sleep fragmentation and decrease the duration of rapid eye movement (REM) and Stage 3 NREM sleep (Mashour and Hudetz, 2017). These disturbances in the sleep–wake cycle promote CRD.

Anesthesia may also contribute to circadian disruption by directly shifting the central pacemaker. General anesthesia has profound effects on the neurotransmitter systems, primarily those mediated by GABA, that regulate the circadian clock. Anesthetic agents such as isoflurane have been shown to exert a phase-shifting effect on the clock by activating SCN GABA receptors (Chong et al., 2021). Furthermore, anesthesia alters the expression of several core clock genes, notably *PER2*. Mori et al. describe how sevoflurane anesthesia suppressed *PER2* expression in the mouse SCN, possibly by reducing histone acetylation in the promoter region (Mori et al., 2014). Interestingly, Gökmen et al. found that daytime isoflurane administration suppressed the expression of clock genes *BMAL1*, *CLOCK*, and *CRY2* in addition to *PER2.* These genes were suppressed in both SCN tissue and peripheral clocks located in the liver (Gökmen et al., 2017).

CRD can be observed in clock output rhythms, such as melatonin. Melatonin is a hormone produced at night and in darkness and serves as a nighttime messenger to the body. The SCN regulates the timing of melatonin secretion through a multi-synaptic pathway extending to the pineal gland. This signaling is dependent on photic information relayed to the SCN from the retina: during the biological night and in darkness, the SCN activates melatonin synthesis, while during the biological day and in light, the SCN inhibits melatonin production (Brown, 1994). Exposure to sufficient light at night ceases melatonin secretion and disrupts sleep (Rea et al., 2020). Surgery has been shown to alter several characteristics of the melatonin rhythm. Gögenur et al. report that the rhythm of the melatonin metabolite AMT6s was delayed after major surgery and that the magnitude of the delay was proportional to the duration of surgery (Gögenur, 2010). In addition, Cronin et al. found that the amplitude of the melatonin rhythm decreased significantly on the first postoperative night (Cronin et al., 2000) and plasma melatonin concentration has been shown to decline after surgery (Shen et al., 2020). Given that melatonin feeds back to the molecular clock by activating the MT (1) and/or MT (2) melatonin receptors of the SCN (Dubocovich, 2007), melatonin rhythm disruption exacerbates CRD, creating a vicious cycle. Similarly, abnormal secretion of the hormone cortisol is highly prevalent among patients undergoing cardiac surgery. Lanuza found that plasma cortisol levels were significantly elevated after both coronary artery bypass grafting and defibrillator implantation surgery (Lanuza, 1995). McIntosh et al. report that major surgery causes a phase shift in the cortisol rhythm (Mcintosh et al., 1981). In some cases, the cortisol rhythm is abolished entirely. Gögenur et al. found that cortisol secretion maintained circadian rhythmicity in 8 out of 11 patients before major surgery, and only 4 patients after surgery (Gögenur et al., 2007b). These disturbances may also reflect circadian misalignment. Just as the SCN regulates cortisol secretion, cortisol levels in turn influence circadian rhythms through glucocorticoid response elements (GREs) located in the peripheral circadian clocks (Nicolaides et al., 2014). GRE-dependent pathways allow glucocorticoids to temporarily phase shift the expression of various peripheral circadian clock genes, notably *Per1* and *Per2*, in heart, liver, and kidney tissue. Dysregulation of the cortisol rhythm thus feeds back to the peripheral clocks, further disrupting circadian rhythms.

### 3.2 Circadian disruption promotes POCD

Perioperative circadian disruption is closely linked to POCD. Patients experiencing sleep deprivation or disruption of sleep–wake rhythms are at significantly higher risk for POCD (Gögenur et al., 2007a; Van Rompaey et al., 2012). In addition, abnormal rhythms of circadian biomarkers, such as melatonin and cortisol, often predict the onset of POCD (Wu et al., 2014; Androsova et al., 2015). Conversely, the administration of melatonin to resynchronize circadian rhythms alleviated symptoms of POCD in murine models (Fan Y. et al., 2017; Song et al., 2018). To explain the correlation between circadian sleep disruption and POCD, two closely interacting pathways have been described in the literature: neuroinflammation and glymphatic dysfunction.

Sleep–wake disruption provokes neuroinflammation even in otherwise healthy individuals by activating microglia (Huang et al., 2014), upregulating complement proteins (Wadhwa et al., 2019), and promoting cytokine production (Wang et al., 2021). This heightened immune activity increases blood brain barrier (BBB) permeability, which results in neuroinflammation and widespread neuronal damage (Cuddapah et al., 2019). Similarly, several studies demonstrate that sleep disruption in a perioperative setting significantly increases levels of circulating cytokines and damages the BBB, causing cognitive dysfunction (Ni et al., 2019; Lu et al., 2020). The inflammatory response to sleep deprivation is exacerbated by the body’s innate immune response to surgical trauma. Invasive cardiac surgery may be complicated by tissue damage, hemorrhage, or ischemia–reperfusion injury, pathological processes that activate inflammation. During procedures involving cardiopulmonary bypass (CPB), a technique that diverts blood away from the heart and lungs, contact between the foreign surface of the CPB circuit and the blood may also cause inflammation (Warltier et al., 2002). This peripheral immune response, coupled with the inflammatory burden of sleep deprivation, results in extensive postoperative neuroinflammation that is thought to play an essential role in the pathogenesis of POCD (Luo et al., 2019).

In addition, perioperative sleep–wake disruption prevents the glymphatic pathway from clearing potentially neurotoxic metabolic waste products from the interstitial space. The glymphatic system is highly active during sleep and suppressed during periods of wakefulness (Jessen et al., 2015; Hablitz et al., 2020). The clearance of the waste in the brain mainly occurs during slow wave sleep, and the influx of cerebrospinal fluid (CSF) is suppressed during wakefulness (Hablitz et al., 2019). Therefore, even brief periods of sleep deprivation can cause significant glymphatic dysfunction and promote the accumulation of harmful compounds in the interstitial fluid (ISF) or brain tissue. Indeed, Shokri-Kojori et al. found that one night of sleep deprivation increased amyloid-β deposition in the hippocampus (Shokri-Kojori et al., 2018), and Holth et al. report that chronic sleep deprivation increases cerebrospinal fluid (CSF) and ISF tau (Holth et al., 2019). Similar effects have been observed in a postoperative setting. Wan et al. show that major surgery is associated with the accumulation of amyloid-β and hyperphosphorylated tau (Wan et al., 2010), and Evered et al. report that levels of tau protein increased significantly in patients after surgery (Evered et al., 2018), indicating glymphatic dysfunction. The abnormal aggregation and buildup of amyloid-β and tau is closely associated with cognitive decline and has been implicated in a wide range of neurodegenerative diseases, including Alzheimer’s disease (Bos et al., 2018). The correlation between these neurotoxic proteins and POCD has been established by the literature: higher levels of amyloid-β (Požgain et al., 2022) and tau (Ramlawi et al., 2006) after surgery have been shown to predict postoperative cognitive impairment. Glymphatic dysfunction therefore represents a viable pathway linking postoperative sleep disruption to POCD.

However, it should be noted that this pathway is influenced by additional factors, such as anesthesia. The effects of general anesthesia on the glymphatic system are complex and multifaceted, as different anesthetic agents have been shown to alternately enhance and suppress glymphatic activity. Several studies have found that the anesthetic dexmedetomidine mimics the effects of slow-wave sleep and thus drives glymphatic activity (Lilius et al., 2019; Persson et al., 2022). Other studies show that general anesthesia impairs glymphatic function by disrupting cerebral arterial pulsatility and respiration, processes that drive CSF-ISF exchange. General anesthesia alters these parameters by suppressing blood pressure and heart rate and substituting positive-pressure mechanical ventilation for spontaneous respiration. Indeed, isoflurane anesthesia was shown to inhibit CSF circulation and glymphatic activity, particularly at high doses (3%) (Gakuba et al., 2018). Hablitz et al. correlated influx of a CSF tracer with electroencephalogram (EEG) power and showed that ketamine/xylazine (K/X) showed the highest CSF tracer influx, while alpha-chloralose, Avertin, or pure isoflurane exhibited the lowest CSF tracer influx when mice were under anesthesia (Hablitz et al., 2019). Future research should clarify the effects of anesthesia on glymphatic function and explore the possibility of a common mechanism to explain these conflicting results.

## 4 Hospital light interventions for sleep and circadian health

Light is the primary *zeitgeber* for the circadian pacemaker, synchronizing a host of physiological processes to the external day-night cycle. Through this pathway, therapeutic light interventions may reset abnormal circadian rhythms in surgical patients, mitigating some of the symptoms associated with POCD.

While there are no formal recommendations for daytime circadian-effective light exposure for hospital environments, recommendations for day-active people have been proposed (UL Standards and Engagement, 2019). In general, the recommendation is to deliver at least 350–500 lux at the eye level during the daytime. Studies showed that hospital rooms or intensive care units (ICU) often lack a robust light–dark pattern, with lighting conditions below the recommended daytime light and too much evening/nighttime light. Tan et al. report average ambient ICU daytime light levels of 100 lux, significantly lower than those recommended by UL 24480 (Tan et al., 2019). Fan et al. showed that light levels in the ICU peaked in the late morning, but the median level was less than 65 lux and light levels never exceeded 150 lux (Fan E. P. et al., 2017). Meyer et al. showed that light levels at night in the ICU were low for the most part, but that at times brief exposures to high light levels at night (>1,000 lux) occurred (Meyer et al., 1994). Verceles et al. measured lights near ICU patients’ beds and showed that light levels were between 40 lux during the daytime and around 2 lux during the nighttime (Verceles et al., 2012). This environment delivering low light levels during the day may prevent patients from maintaining stable circadian rhythms (Durrington et al., 2017). In fact, the lack of circadian-effective daytime light has been shown to be associated with decreased circadian rhythm amplitude (Bano-Otalora et al., 2020), poor sleep quality, and delayed sleep onset (Blume et al., 2019). Bernhofer et al. also found that low daytime light levels in hospitals predicted sleep fragmentation and mood disturbances among patients (Bernhofer et al., 2014).

Life has evolved around the 24-h cycle of robust light and darkness that constitutes Earth’s solar day. Hospital lighting should therefore mirror this robust light–dark cycle, creating a clear distinction between day and night. Daylight is the ideal light source for the circadian system and can be delivered to hospital rooms via windows or skylights. However, this option is not always possible in hospital facilities. When that is the case, electric lighting to deliver circadian-effective light during the day can be used (Knoop et al., 2020; Münch et al., 2020; Wirz-Justice et al., 2021). A general model or “target” for circadian-effective lighting has been established in the literature (Rea and Figueiro, 2018; UL Standards and Engagement, 2019; Rea et al., 2021a,b). Lighting interventions providing circadian-effective light exposures have shown great promise as a nonpharmacological treatment to help regulate sleep in populations at risk for circadian rhythm disruption, such as persons living with Alzheimer’s disease and related dementias. Studies have demonstrated that daytime light exposure can consolidate and increase nighttime sleep efficiency, while increasing daytime wakefulness and reducing evening agitation in this population (Figueiro et al., 2014, 2015, 2016, 2019, 2020; Figueiro, 2017; Rubiño et al., 2017, 2020). Similar light therapy approaches have been shown to improve mood outcomes, perhaps via improved circadian entrainment, in patients undergoing autologous stem cell transplantation hospitalization for the treatment of multiple myeloma (Valdimarsdottir et al., 2018). Light therapy has also shown promise as a means for improving circadian entrainment and outcomes in the clinical management of genitourinary cancers (Kaur et al., 2022). There is no reason to presume that light therapy could not also be used to improve outcomes in patients experiencing perioperative circadian disruption and POCD resulting from cardiac surgery.

Indeed, the implementation of circadian-effective lighting in hospitals has been shown to improve sleep quality and reduce the incidence of cognitive dysfunction. Wakamura and Tokura found that 5 h of daytime exposure to bright light significantly increased both sleep duration and melatonin secretion among hospitalized patients (Wakamura and Tokura, 2001). In addition, bright light therapy was associated with reduced risk of delirium among surgical ICU patients (Potharajaroen et al., 2018). Decreased nocturnal light levels had similar effects on circadian rhythmicity. Patel et al. found that reducing ambient light and noise at night improved patients’ sleep quality and decreased delirium incidence in the ICU (Patel et al., 2014). Circadian-effective lighting has been established as an effective tool to re-synchronize abnormal rhythms to the natural day-night cycle, thus preventing sleep disruption and cognitive decline.

## 5 Discussion

In an environment where numerous factors threaten the stability and rhythmicity of the circadian clock, lighting interventions represent a route toward reducing CRD and cognitive dysfunction in cardiac surgery patients. Circadian disruption is common among patients undergoing cardiac surgery due to sleep deprivation, inflammation, and the suppressive effects of anesthesia on several clock genes. These disturbances manifest as changes in the rhythmic secretion of circadian markers, such as melatonin and cortisol, and feed back to exacerbate sleep–wake disruption (Gögenur et al., 2007b). Circadian misalignment has immediate and profound consequences for cognitive function in surgical patients. On a molecular level, sleep deprivation incites harmful neuroinflammation and oxidative stress, resulting in tissue damage (Lu et al., 2020; Wang et al., 2021). On a broader physiological level, circadian disruption reduces hippocampal volume, disturbs the structural integrity of the BBB, and impairs glymphatic system function (Ni et al., 2019). These neurocognitive complications may cause long-term damage and slow the postoperative recovery process for patients. Therefore, re-synchronizing abnormal circadian rhythms to the natural day-night cycle may help address the pathogenesis of POCD. Because the circadian pacemaker is directly regulated by photic information emanating from retinal photoreceptors, therapeutic lighting interventions provide a non-invasive, non-pharmacological means of entraining circadian rhythms in surgical patients. There is growing evidence that circadian-effective lighting, characterized by bright daytime and dim nighttime light, effectively restores normal rhythms of circadian markers and corrects the sleep–wake cycle. These effects may extend beyond the circadian pacemaker to reduce the delirium burden in patients undergoing major surgery (Potharajaroen et al., 2018). Future research should focus on establishing optimal parameters for circadian-effective lighting while navigating the need for visibility in a hospital setting.

## Author contributions

EC: Writing – original draft. MF: Conceptualization, Funding acquisition, Methodology, Project administration, Writing – review & editing.
