# Corrigendum: Postoperative cognitive dysfunction: spotlight on light, circadian rhythms, and sleep

**DOI:** 10.3389/fnins.2024.1419709

**Published:** 2024-05-06

**Authors:** Ellie Campbell, Mariana G. Figueiro

**Affiliations:** Light and Health Research Center, Department of Population Health Science and Policy, Icahn School of Medicine at Mount Sinai, New York, NY, United States

**Keywords:** cardiac surgery, circadian rhythm, circadian rhythm disruption, cognitive dysfunction, sleep

In the published article, there was an error. A paragraph from Section 2.2 was mistakenly duplicated in Section 2.1. **Section 2.1**, *The circadian system*, paragraph 3 previously stated:

“Clock genes, particularly the *PER2* gene, have also been associated with the homeostatic process of sleep. A common variant in *PER2* was shown to be associated with a 20-min reduction in slow wave sleep, which is a marker of sleep homeostasis (Chang et al., 2016). Those with a polymorphism in the *PER3* clock gene (*PER3*(5/5)) tend to be morning types and exhibit more rapid build-up of sleep pressure during sleep deprivation. These studies show the close link between clock genes and the sleep–wake cycle.”

This duplicate paragraph has now been removed from Section 2.1.

The authors apologize for this error and state that this does not change the scientific conclusions of the article in any way. The original article has been updated.

